# Heterozygous Nonsense Mutation in the Nuclear Transport Factor *KPNA7*, a Maternal Factor Active in Embryonic Tissues, Causes Autosomal Dominant Otosclerosis

**DOI:** 10.3390/ijms27114985

**Published:** 2026-05-30

**Authors:** Tammy Benteau, Nelly Abdelfatah, Anne Griffin, Cindy Penney, Pingzhao Hu, Susan G. Stanton, Guangju Zhai, Maxime Maheu, Curtis R. French, Terry-Lynn Young

**Affiliations:** 1 Division of Biomedical Science, Faculty of Medicine, Memorial University, St. John’s, NL A1B 3V6, Canada; tbenteau@mun.ca (T.B.); nelly.abdelfatah@kingstonhsc.ca (N.A.); gzhai@mun.ca (G.Z.); v77crf@mun.ca (C.R.F.); 2 Centre for Translational Genomics, Craig L. Dobbin Genetics Research Centre, 300 Prince Philip Drive, St. John’s, NL A1B 3V6, Canada; 3 Department of Biochemistry and Computer Science, Western University, London, ON N6G 2V4, Canada; phu49@uwo.ca; 4 National Centre for Audiology & School of Communication Sciences and Disorders, Faculty of Health Sciences, Western University, London, ON N6G 1H1, Canada; sstanto2@uwo.ca; 5 École d’Orthophonie et d’Audiologie, Université de Montréal, Montréal, QC H3C 3J7, Canada; maxime.maheu.1@umontreal.ca

**Keywords:** KPNA7, RELN, otosclerosis, autosomal dominant, *OTSC*, conductive hearing loss, globuli interossei, ncNLS transport, Importin-α, Importin-β

## Abstract

Otosclerosis is a common cause of conductive hearing loss thought to result from dysregulated bone remodeling in the embryonic tissues of the globuli interossei. Both familial and sporadic cases have been reported. To date, 10 published *OTSC* loci and four genes (*FOXL1* (*OTSC11*), *SMARCA4* (*OTSC12*), *MEPE*, *SERPINF1*) have been identified in autosomal dominant families. Using a combined genetic and genomics approach in five affected siblings, we identified a nonsense mutation in Karyopherin subunit α7 (*KPNA7*, c.49C>T, p.R17X), the newest of the importin-α family of nuclear transporters. KPNA7 is a key maternal factor involved in the classical transport of NLS-containing cargo proteins, active during early embryonic cleavage events and zygotic genome activation. So far, 377 cargo proteins associated with KPNA7 have been identified. Recessive *KPNA7* variants cause skeletal abnormalities, epilepsy, intellectual disabilities and preimplantation embryo arrest (PREMBA). A closer look at the *OTSC* genes reveals their involvement in endochondral ossification signaling pathways. We explore how KPNA7 haploinsufficiency in the embryonic tissues of the otic capsule may cause dysregulated bone remodeling. This study expands the phenotypic spectrum of KPNA7 and provides new insights into the pathobiology of otosclerosis.

## 1. Introduction

Otosclerosis is a uniquely human skeletal disorder restricted to the otic capsule of the temporal bone and a common cause of progressive conductive hearing loss (HL) in young adults. Otosclerosis is clinically characterized by abnormal bone deposition (sclerotic lesions) in the middle ear, causing the fine structures of the ossicles, especially the stapes bone, to become distorted and limiting the movement of the stapes footplate against the oval window. Eventually, the immobilization of the stapes bone results in conductive HL, as well as sensorineural HL in some patients if new bony deposits extend into the fluid-filled inner ear [1]. The osseous labyrinth protects the delicate tissues, structures and spaces of the middle/inner ear but poses challenges to perilymph sampling for diagnostic purposes and local drug delivery. Visualization during surgery to replace or repair stapes fixation validates the diagnosis, and in most cases, surgery restores the conductive component of hearing to those able to access stapes surgical prosthesis replacement (Figure S1). Otosclerosis risk factors include positive family history, sex (female), measles and pregnancy [1,2]. Recognized as a medical entity some 125 years ago [3], otosclerosis cannot be predicted, stopped or medically treated, and its pathogenesis remains a mystery.

Otosclerosis is currently considered an autosomal dominant (AD) disease with environmental triggers [1]. Despite several decades of research, the otosclerosis (*OTSC*) genes have been recalcitrant to discovery, likely due to the genetically heterogeneous nature of otosclerosis and the rarity of AD families under study [2]. To date, 10 published *OTSC* loci and four genes, *FOXL1* (*OTSC11*), *SMARCA4* (*OTSC12*), *MEPE*, and *SERPINF1*, have been identified in AD families (Hereditary Hearing Loss Homepage, http://hereditaryhearingloss.org/, accessed on 11 March 2026). We reported the first *OTSC* gene, *FOXL1*, in two Canadian families and noted delayed mineralization in the jaw bones (ossicle equivalents) of a *foxl1* mutant zebrafish strain [4,5]. A recent GWAS of 3504 otosclerosis cases revealed 23 novel loci in close physical proximity to genes whose dysregulation in bone remodeling and mineralization causes rare monogenic skeletal disorders [6]. In most association loci, the nearest protein-coding genes provide insight into the nature of the highly penetrant *OTSC* genes but not their identity, as GWAS design excludes rare variants. Given the high genetic heterogeneity underlying skeletal dysplasias, with 461 genes known to cause monogenic forms [7], more *OTSC* genes can be anticipated.

A closer look at the *OTSC* genes *SMARCA4*, *FOXL1*, *SERPINF1* and *MEPE* reveals their involvement in endochondral ossification signaling pathways. SMARCA4 (also known as BRG1) plays a role in ossicle formation during embryogenesis and may be important for regulation of osteoblast differentiation and maintenance of postnatal bone homeostasis in the otic capsule [8]. We demonstrated that foxl1 in a zebrafish model of otosclerosis regulated the expression of collagen genes *col1a1* and *col11a2* and resulted in a delay in jawbone mineralization [4]. *SERPINF1* encodes for PEDF, which is involved in many biological processes, including bone formation. SERPINF1 binds to extracellular matrix proteins, including collagen and glycosaminoglycan, and may be involved in the mineralization of the bone matrix [9]. MEPE normally serves as a decoy receptor for pre-osteoclasts, inhibiting osteoclast maturation, and its ASARM motif, upon proteolytic cleavage by β-cathepsin, inhibits mineralization by binding to hydroxyapatite crystals [10].

Herein we identify a nonsense mutation in Karyopherin subunit α7 (*KPNA7*) causing otosclerosis in a white Canadian family of Northern European extraction. *KPNA7* encodes the newest of the seven human Importin-α (Imp-α) transport adaptor molecules. Nucleocytoplasmic trafficking involves the active transport of large proteins (>60 k Daltons) containing a nuclear localization signal (NLS) across the nuclear membrane. Dysregulation of this highly efficient transport system is linked to major diseases such as cancer, viral infections, inflammation and neurodegenerative diseases, making it a prime target for therapies [11]. The Imp-αs serve as adaptor molecules in classical (cNLS) transport, where they bind cargo proteins and Importin-β (Imp-β), the carrier molecule that transports the tricomplex (cargo-Imp-α-β) across the nuclear membrane. In non-classical (ncNLS) transport, Imp-β binds directly to cargo protein without the need for an Imp-α adaptor. Imp-αs can also inhibit the ncNLS transport of some proteins [12]. KPNA7 is the most structurally divergent of the Imp-α adaptors, is expressed during early development and is increasingly recognized as a key maternal factor essential for embryogenesis and fertility. Recessive *KPNA7* mutations cause preimplantation embryo arrest (PREMBA) [13] and developmental disability in concert with skeletal abnormalities [14]. *KPNA7* is an intriguing *OTSC* gene, and we explore how this embryonically expressed gene may cause adult-onset HL and garner insights into the pathobiology of otosclerosis.

## 2. Results

### 2.1. Pedigree Structure and Classification of Otosclerosis

**Figure 1:**
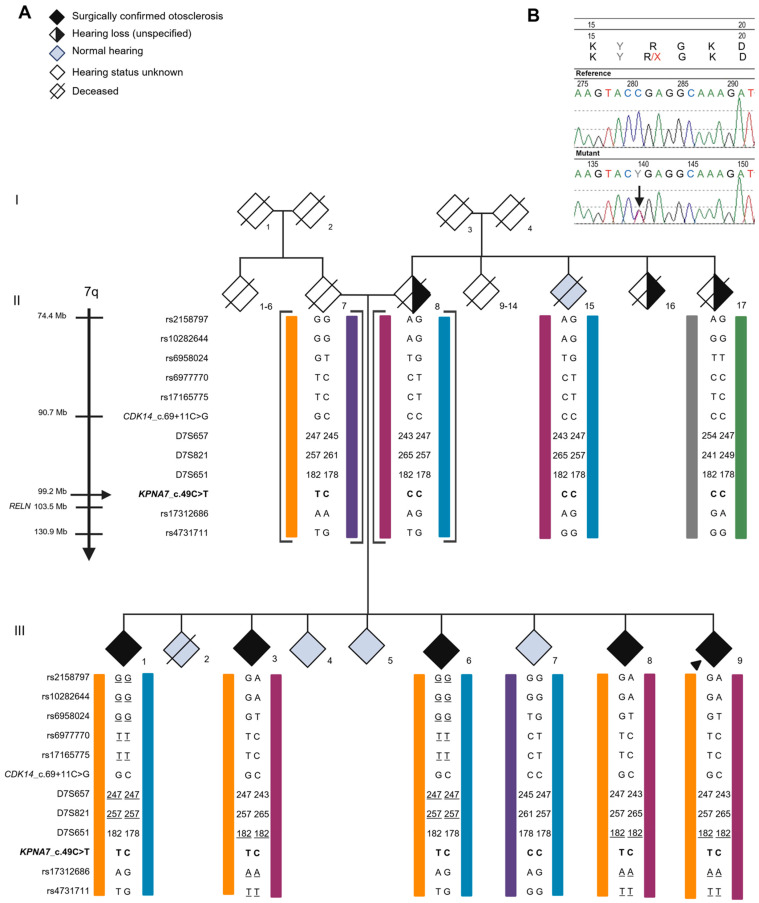
Pedigree of a multiplex family from the island of Newfoundland, Canada with clinically confirmed otosclerosis. PID III-9 is the proband (depicted by arrow below the symbol). (**A**) Otosclerosis-associated haplotype covering 56.5 Mb on chromosome 7q. To recapitulate the otosclerosis-associated haplotype, given the deceased parents (no DNA; unknown otosclerosis status), we first phased only the markers that were homozygous in one or more of the affected siblings (alleles underscored) and adhered to the rule of the least number of recombinations. We could be confident in the recapitulated haplotypes, as no recombinations were invoked. The orange, paternally derived haplotype carries the *KPNA7*, c.49C>T, p.R17X mutation (bolded). Diamonds were used to protect identity. (**B**) Sequencing electropherogram showing *KPNA7*, c.49C>T, p.R17X (Mutation Surveyor software, version 5.00, SoftGenetics LLC, State College, PA 16803, USA), where genomic positions were captured using GRCh38/hg38. Created in BioRender. BENTEAU, T. (https://BioRender.com/dm8akgi, accessed on 22 May 2026) is licensed under CC BY 4.0.

**Figure 2:**
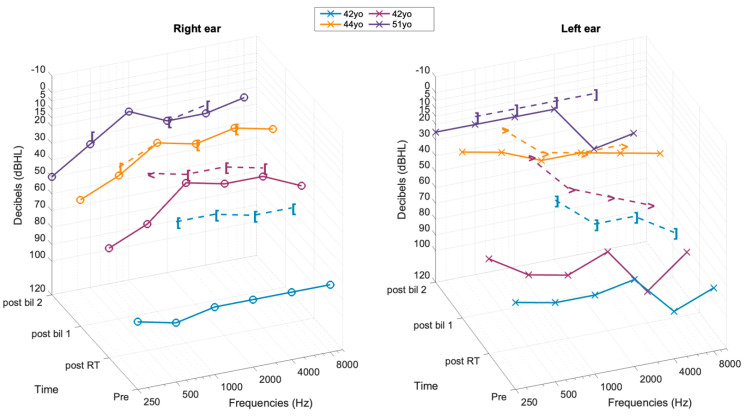
Pre- and post-stapedectomy audiograms reveal successful surgical treatment of bilateral otosclerosis in the proband’s sibling, PID III-1. Solid lines represent air conduction and dashed lines represent bone conduction. Pre = pre-stapedectomy audiogram, age 42; post RT = post-stapedectomy audiogram, right ear, age 42; post bil 1 = first post-bilateral stapedectomy audiogram, age 44; post bil 2 = second post-bilateral stapedectomy audiogram, age 51; ○ = right ear unmasked air conduction; × = left ear unmasked air conduction; [ (right) and ] (left) = unmasked bone conduction; and < (right) and > (left) = masked bone conduction (https://www.mathworks.com/, accessed on 25 July 2025).

The proband (PID III-9) is one of nine siblings, five diagnosed with otosclerosis, whose parents are deceased. Little is known about the parental generation. The father (PID II-7) reportedly had normal hearing but was “very sensitive to noise.” The mother (PID II-8), along with two (PIDs II-16, II-17) of her siblings, had uncategorized HL. X-linked inheritance could be ruled out, as otosclerosis is not more severe in males; however, the pedigree was consistent with both AD and AR (autosomal recessive) inheritance, as we were unable to confirm otosclerosis in either of the deceased parents (Figure 1). Surgical reports documenting the visualization of stapes fixation confirmed the diagnosis of otosclerosis in the five affected siblings. HL was first noted during adolescence in four siblings and early adulthood in the fifth. Stapedectomy surgery restored hearing in all affected. Audiograms post-stapedectomy were not available for the proband. However, a complete pre- and post-series of audiograms was available on PID III-1 (Figure 2). PID III-1 was diagnosed at 42 with severe, bilateral HL due to otosclerosis, which was noted in their teenage years (Figure 2). A pre-surgery audiogram showed conductive loss with borderline cochlear (sensorineural) HL. Post-stapedectomy of the right ear improved hearing, and the air–bone gap was mainly resolved, but some HL remained, especially in the low frequencies. After stapedectomy of the left ear at age 44, hearing improved across all frequencies, with only mild sensorineural HL remaining in the mid- to high frequencies. At age 51, PID III-1 experienced mild HL in the low and mid-frequencies of the left ear, but high frequencies showed moderate to severe loss at 4000 Hz and 8000 Hz, likely due to disease processes on the cochlear side of the round window.

### 2.2. Haplotype Exclusion/Targeted Screening at OTSC Loci and Otosclerosis-Associated Genes

To identify potential overlap of otosclerosis in this family to known *OTSC* genes/loci, we genotyped informative microsatellite markers and phased alleles to look for haplotype sharing among the five affected siblings in accordance with both AR and AD inheritance patterns. Analysis of haplotype sharing ruled out *OTSC1* (15q), *OTSC3* (6p), *OTSC4* (16q), *OTSC5* (3q), *OTSC7* (6q), *OTSC8* (9p), *COL1A1* (17q) and *NOG* (17q). Furthermore, the proband screened negative for the *FOXL1* deletion (rs764026385; *OTSC11*) and the otosclerosis-associated variants in *SERPINF1* [15]. However, partial haplotype sharing in the vicinity of the *OTSC2* locus under the AD model was noted (Figure S2). Haplotype exclusion ruled out linkage to known *OTSC* loci and two otosclerosis-associated genes.

### 2.3. Genome-Wide SNP Genotyping, Linkage and WES Under Suggestive Genomic Region(s)

To identify the genomic loci shared between all affected siblings, we carried out genome-wide SNP genotyping and linkage analyses under both AR and AD inheritance models. We genotyped the five affected siblings (PIDs III-1, III-3, III-6, III-8, and III-9), an unaffected sibling (PID III-7), a maternal sibling with HL (PID II-17) and a maternal sibling with normal hearing (PID II-15) (Figure 1). Under an AR model and given the pedigree structure, linkage simulation derived a theoretical maximum LOD score (LOD_max_) of 2.5. Multipoint linkage analysis yielded the LOD_max_ at chr17q25.1-q25.3, containing 141 annotated genes (5.9 Mb). WES revealed no homozygous and 49 heterozygous variants. Of these, only *TEN1* and *EVPL* had two or more variants consistent with AR inheritance. All *TEN1* and *EVPL* variants were subsequently filtered out due to high population frequencies and/or benign functional predictions [15]. Linkage simulation under an AD model derived a LOD_max_ score of 1.73. Suggestive linkage scores (LOD > 1) were observed at five distinct genomic loci (7q, 10p, 10q, 16q, 17q) overlapping *OTSC2* (7q) and *OTSC11* (16q). The region under these five genomic loci contained a total of 542 annotated genes.

To identify potential disease-causing variants within linked/suggestive genomic regions, WES was carried out on four of the affected (PIDs III-1, III-3, III-6, and III-9) and two older population controls (55, 60 years old) with normal hearing thresholds. Sequencing variants were subjected to a multi-step filtering pipeline (Figure S3). The WES revealed >1600 variants across 542 positional candidate genes. However, the vast majority (>80%) were identified in the hearing controls and were removed, with a total of 300 variants remaining. Further filtering removed 153 variants absent in one or more affected, 37 variants identified in solved *FOXL1* cases and 92 variants with MAF ≥ 2%. In silico analyses enabled the filtering of an additional 10 missense variants predicted to be benign and five silent and one intronic variants predicted to not affect splicing (Table S1). A single remaining frameshift variant was filtered out due to non-segregation with otosclerosis in the family.

Only one nonsense variant, *KPNA7*, c.49 C>T (NM_001145715.3; rs746784660), could not be filtered out. Subsequent cascade sequencing of the extended family confirmed co-segregation of *KPNA7*, c.49 C>T in an AD segregation pattern. We did not observe this variant among the 3184 alleles tested (187 HL probands, 1292 population controls, 113 clinical samples). Although we did not have DNA on the parents, haplotype phasing was achieved with the aid of DNA from the maternal siblings (PIDs II-15, II-17), confirming paternal transmission (Figure 1). *KPNA7* resides outside of the published *OTSC2* locus on chromosome 7q [16] but maps within 4.3 Mb of *RELN*, an otosclerosis-associated gene [17] (Figure S2).

### 2.4. KPNA7 In Silico Analyses and ACMG Classification of Pathogenicity

*KPNA7* is a known disease gene; recessive mutations cause congenital skeletal abnormalities [14] and PREMBA [13]. ClinVar classifies *KPNA7*, c.49 C>T as a VUS (ClinVar ID: 652650, accessed 11 March 2026). The *KPNA7* gene contains 11 exons. The c.49C>T (p.R17X) variant lies within exon 2 and introduces a premature termination codon predicted to result in nonsense-mediated mRNA decay and haploinsufficiency, consistent with PVS1 (very strong) evidence for pathogenicity. The *KPNA7* c.49C>T variant co-segregated with otosclerosis in all affected individuals in this family, providing supporting segregation evidence (PP1). *KPNA7* c.49C>T is extremely rare, with a minor allele frequency of 0.01% in gnomAD (accessed 11 March 2026), consistent with PM2 (absence or rarity in population databases). According to the ACMG variant classification guidelines, *KPNA7*, c.49 C>T, p. R17X is pathogenic (PVS1, PM2), with supporting segregation evidence (PP1) [18,19].

## 3. Discussion

### 3.1. Summary of Findings

We identified *KPNA7*, a new *OTSC* gene, in a white multiplex family of Northern European extraction. KPNA7 is the first member of the nucleocytoplasmic trafficking system identified to cause otosclerosis. Surgical reports confirmed the diagnosis, and the affected siblings reported HL as young adults. Challenges in phasing disease-associated alleles due to deceased parents were overcome by recruiting maternal siblings. A comprehensive genetic and genomics approach successfully determined the paternal transmission of a pathogenic nonsense mutation [c.49 C>T, p. R17X] exclusively to five affected siblings. Heterozygous individuals likely developed otosclerosis due to KPNA7 haploinsufficiency. *KPNA7* is an unexpected *OTSC* gene, as it causes a highly penetrant adult-onset conductive hearing loss despite being normally active only during embryogenesis. The globuli interossei, the proposed site of otosclerosis, contains embryonic islands, suggesting KPNA7 may be responsible for maintaining chondrocytes and osteocytes in a quiescent state.

### 3.2. Phenotypic Effects of Mutant KPNA7 During Embryogenesis and Reactivation in Cancer

KPNA7 is the most divergent of the seven human Imp-α adaptor proteins and is involved in cNLS transport and inhibition of ncNLS transport of certain proteins [12]. Interestingly, KPNA7 is expressed during early embryogenesis, with 377 different cargo proteins identified so far [20]. KPNA7 is the most abundant Imp-α adaptor in germinal vesicle and metaphase II-stage oocytes, and it is rapidly degraded during zygotic genome activation, becoming barely detectable in morula- and blastocyst-stage embryos [21,22]. Increasingly recognized as a key maternal factor essential for embryogenesis and fertility, two non-functioning *KPNA7* alleles cause PREMBA due to the failure of embryos to develop past the blastula stage and implant. Postnatally, KPNA7 is absent in non-germinal tissues in normal development. In cancer, genomic amplification of 7q21-22 increases the nuclear import rate of KPNA7 cargo proteins, including cell cycle factors promoting carcinogenesis, due to increases in KPNA7 expression [23].

### 3.3. Endochondral Ossification and Bone Remodeling in the Otic Capsule

The otic capsule has a highly complex anatomy, and its embryonic development is one of the most complicated examples of cellular morphogenesis in any biological system [24]. It forms through endochondral ossification, one of two essential pathways of bone formation that uses cartilage as a template during fetal development. During this process, mesenchymal stem cells differentiate into chondrocytes (cartilage cells), which proliferate rapidly, hypertrophy and secrete the extracellular matrix that undergoes mineralization. Eventually the hypertrophic chondrocytes undergo apoptosis and are replaced by osteocytes that become trapped in the bony matrix. Unlike the rest of the human skeleton, the otic capsule is fully formed by the fifth fetal month [25]. Bone development is arrested at this point; however, islands of quiescent chondrocytes and osteocytes (globuli interossei) are uniquely retained and persist throughout life. It is these globuli interossei that are thought to be the site of otosclerosis [26,27,28].

Postnatally, the human skeleton is maintained and repaired by bone remodeling, a well-known process involving the RANK/RANKL/OPG pathway. However, in the otic capsule, bone remodeling is suppressed due to excess OPG, arresting the process of osteoclastogenesis [29]. Bloch and colleagues have suggested that microdamage accumulating in the aging human perilabyrinthine bone contributes to otosclerosis [30,31,32,33,34,35]. According to this theory, as the perilacunar matrix breaks down, clusters of dead osteocytes trapped in the bony matrix create cellular voids that lose connection with each other, rendering OPG and other nutrients beyond the reach of viable but isolated osteocytes [31]. In the absence of OPG, chondrocytes and osteocytes lying dormant within the globuli interossei would be able to resume the process of endochondral ossification. If this is the case, perhaps it is this new bony deposition that adheres the stapes bone to the oval window, causing conductive HL.

### 3.4. Explorations into the Pathobiology of KPNA7, an Embryonically Expressed Otosclerosis Gene

The preservation of the otic capsule, the hardest bone in the body, is essential to maintaining hearing integrity [36]. Excess OPG is a known protective mechanism against new bone formation operating in the otic capsule, as it arrests the process of bone remodeling [29]. Otosclerosis due to KPNA7 haploinsufficiency may be caused by the inadvertent nuclear import of a growth promotor (via ncNLS transport). For example, Imp-αs have been shown to inhibit the nuclear import of parathyroid hormone-related protein (PTHrP) and SNAIL [12], both of which play important roles in bone growth [29,37,38]. Alternatively, KPNA7 haploinsufficiency may be responsible for the decreased import of a growth inhibitor (via cNLS transport) in quiescent chondrocytes and osteocytes of the globuli interossei. As the full spectrum of KPNA7 cargo proteins is unknown, being the newest member of the Imp-αs, further investigations are warranted. We are currently creating a *kpna7* mutant zebrafish strain to examine the jaw bones (ossicle equivalents) and determine the underlying disease mechanism. We believe that KPNA7 plays a protective role, in addition to OPG, against new bony growth in the mature otic capsule. Given that normal *KPNA7* gene expression is restricted to embryonic tissues, KPNA7 haploinsufficiency would not be expected to cause global skeletal effects outside of the otic capsule, such as osteopetrosis (dysfunctional osteoclasts), osteopoikilosis (bone islands) and multiple diaphyseal sclerosis (ribbing disease), to name a few [39].

### 3.5. A Combined Genetic and Genomic Approach Is Needed to Identify the OTSC Genes

AD conditions are challenging to resolve, particularly when the phenotype is relatively common, as is the case with conductive HL. Based on our experience, successful identification of recalcitrant *OTSC* genes requires three critical elements often lacking in gene discovery studies: (1) surgical confirmation of otosclerosis, (2) iterative genetic and genomic analyses and (3) comprehensive family ascertainment. Although more labor-intensive than GWAS, this strategy effectively addresses genetic heterogeneity, rare pathogenic variants, and phenocopies. In the present study, the absence of parental DNA or clinical information posed additional challenges and initially led to an incorrect assumption of maternal transmission. Recruitment of maternal siblings was essential for accurate haplotype assignment, and phasing of homozygous markers in the affected siblings supported paternal transmission of the otosclerosis-associated haplotype. Finally, two filtering steps, the use of population controls (with normal hearing thresholds) and solved *FOXL1* cases, vastly reduced the number of variants of interest to <10% of the total.

### 3.6. Limitations of This Study and Future Directions

One major limitation in this study is the lack of functional studies. According to ACMG guidelines, the *KPNA7* (p.R17X) premature stop mutation is pathogenic, and the evidence strongly suggests that p.R17X is the cause of the AD otosclerosis in this white family of Northern European descent. *KPNA7* represents the second *OTSC* gene identified in the white settler population of the island of Newfoundland. In the absence of patient-derived disease tissue, equal expression of parental *KPNA7* alleles is also assumed. Functional studies, including hypothesis-driven animal models, are needed to elucidate the pathogenic mechanism, including whether KPNA7 haploinsufficiency leads to reactivation of endochondral ossification or an alternative process.

We encourage renewed efforts to identify genes underlying published *OTSC* loci through rigorous phenotypic confirmation of otosclerosis, iterative genetic and genomic analyses, comprehensive family ascertainment and functional analysis of variants of interest. As well, non-coding RNA, the class of molecules that regulate gene expression, is increasing recognized for its role in cellular processes, and screening of mapped *OTSC* genomic loci should include positional non-coding RNA genes. Recent advances in microneedle technologies now enable safe sampling of inner ear fluids, overcoming long-standing barriers to diagnosis and targeted therapy by allowing microliter-scale perilymph collection [40]. Together, identification of *OTSC* genes and improved access to inner ear biology will facilitate the discovery of environmental triggers, transforming otosclerosis from an idiopathic condition into a predictable and treatable disease. Our findings establish *KPNA7* as a novel gene underlying AD otosclerosis and provide new insight into the role of nuclear transport in otic capsule bone remodeling.

## 4. Materials and Methods

### 4.1. Ethics Statement

Prior approval to study live research participants with HL and their blood relatives was granted by the regional ethics authority on 4 April 2002 (Hearing Loss Project #01.186, Human Research Ethics Board, St John’s, NL, Canada), and research participants provided written informed consent.

### 4.2. Pedigree Structure and Clinical Classification of Otosclerosis

Research participants underwent audiological and medical examinations, permitted access to their medical records, and completed a medical questionnaire [15]. HL was classified based on the pure-tone threshold averages of 0.5, 1.0 and 2.0 kHz, as defined by the American Speech and Hearing Association (ASHA) (https://www.asha.org/practice-portal/clinical-topics/hearing-loss/, accessed on 30 May 2025). Family members were assessed by our clinical team to update audiograms and confirm middle ear status. A difference of >10 dB HL between air and bone conduction sensitivity represented a significant conductive component associated with impaired sound transfer through the middle ear. Conservative clinical criteria were used to assign otosclerosis affection status: the affected were blood relatives with surgically confirmed otosclerosis at any age; the unaffected were blood relatives ≥ 55 years of age with normal bilateral hearing thresholds.

### 4.3. Genotyping, Hapolotyping and Targeted Gene Sequencing

Genomic DNA on the extended family was extracted from peripheral blood [41]. The parents were deceased and not available to study. Informative microsatellite markers (4–10 per locus) spanning seven *OTSC* loci, *OTSC1* (15q), *OTSC2* (7q), *OTSC3* (6p), *OTSC4* (16q), *OTSC5* (3q), *OTSC7* (6q), *OTSC8* (9p), and the otosclerosis-susceptibility genes *COL1A1* (17q) and *NOG* (17q) were genotyped, and allelic sharing was assessed among the five affected siblings. Primers were labeled (6-FAM), amplified using touchdown PCR, size-fractionated (ABI PRISM model 3130xl) and analyzed with GeneMapper software (v4.0). Alleles were phased and haplotypes recapitulated according to “least recombination rules.” As the parents are deceased, parental haplotypes were inferred. *OTSC* loci were excluded if a shared haplotype was not identified across all five affected siblings. We also carried out targeted Sanger sequencing of otosclerosis variants, including the 15 bp coding deletion in *FOXL1* (*OTSC11*; rs764026385) and rare associated variants in *SERPINF1* [42] [March 2006 assembly (NCBI build 36.1)].

### 4.4. Genome-Wide SNP Genotyping and Linkage Analyses

Given the ambiguous inheritance pattern (due to deceased parents), genome-wide SNP genotyping and multipoint linkage analysis were performed under both AR and AD models. We used Merlin (version 1.1.2) [43] and assumed complete (100%) penetrance. LOD scores were calculated at recombination fractions of 0.000 to 0.5000. We genotyped the 610K Illumina SNP array (McGill Genome Centre, McGill University, QC, Canada) on samples from five affected siblings (PIDs III-1, III-3, III-6, III-8, III-9), an unaffected sibling (PID III-7), a maternal sibling with HL (PID II-17) and a maternal sibling with normal hearing (PID II-15) (Figure 1). As well, nine population control samples with normal hearing were used to estimate the minor allele frequencies of unavailable family members. Genotypes were analyzed at The Centre for Applied Genomics (TCAG, University of Toronto, ON, Canada) and exported from GenomeStudio software (v2010.3).

### 4.5. WES, Variant Filtering and Cascade Sequencing Under New Linked Region(s)

WES was performed at the McGill Genome Centre (September 2012). Libraries were prepared using the TruSeq preparation kit and sequenced on an Illumina HiSeq 2000 platform, generating 50–150 million 100 bp paired-end reads per sample. Reads were trimmed from the 3′ end to retain bases with Phred quality scores ≥30; adapters were removed and reads shorter than 32 bp were excluded. Filtered reads were aligned to the 1000 Genomes human reference genome using the Burrows–Wheeler Aligner (BWA). Alignments were generated per sequencing lane and merged using Picard. Local realignment around insertions and deletions was performed using GATK to reduce false-positive calls caused by misalignment in regions where multiple mismatches may otherwise have been preferred over indels by the aligner [44]. Mate-pair information was recalculated with Picard, and PCR/optical duplicates were marked using Picard, retaining the best duplicate pair based on alignment score.

Variant calling was performed using samtools mpileup followed by bcftools varfilter, as implemented in the McGill Genome Centre/Genome Québec exome pipeline, with detailed command-line parameters following the facility protocol [45]. Variant-level quality control was applied to reduce low-confidence calls generated during the variant-calling pipeline. Calls were evaluated using site-level and sample-level metrics, including variant quality, read depth, mapping quality, strand bias, genotype likelihood, genotype quality, and mappability annotations. Variants in regions flagged as high-coverage (>400×), low-coverage (<50×), low-mean mapping quality (<20), or no-data were interpreted cautiously or were not prioritized.

WES was carried out on four affected (PIDs III-1, III-3, III-6, and III-9) and two older population controls (55, 60 years old) with normal hearing thresholds. Sequencing variants were subjected to a multi-step filtering pipeline (Figure S3). Downstream analysis focused on variants with ≥20× coverage and minor allele frequencies <2%, the latter threshold allowing for possible founder effects. For candidate heterozygous variants, allelic balance was assessed using alternate-read fractions relative to total read depth. Calls with insufficient alternate-allele support, strong allelic imbalance, poor genotype quality, strand bias, poor mapping quality, or inconsistent segregation among affected individuals and unaffected controls were not considered validated. Candidate variants were further assessed using UCSC Genome Browser and/or Alamut Visual Plus for pathogenicity prediction, including REVEL scores for missense variants and splice-site prediction for synonymous, frameshift, and intronic variants [46]. The *KPNA7* nonsense variant was tested against 38 otosclerosis probands (from ON, Canada; Western University ethics #103,679); 149 HL probands; 1292 population controls; and 113 clinical samples. The 1292 population controls represented 157 research samples; 693 NL Osteoarthritis Study (NFOAS) patients and 442 NL Colorectal Cancer Registry patients (genotyped by Illumina microarray platforms and then imputed with 1000 genome project data as reference panels). Pathogenicity was assessed by ACMG criteria for hearing loss variants [18,19].

## Data Availability

The original contributions presented in this study are included in the article/Supplementary Material. Further inquiries can be directed to the corresponding author. The SNVs are available in the dbSNP repository (https://www.ncbi.nlm.nih.gov/snp/, accessed on 15 October 2025).

## Supplementary Materials

The following supporting information can be downloaded at: https://www.mdpi.com/article/10.3390/ijms27114985/s1.

## Abbreviations

The following abbreviations are used in this manuscript:

ACMG	American College of Medical Genetics
AD	Autosomal dominant
AR	Autosomal recessive
ASARM	Acidic, serine– and aspartate-rich motif
BP	Base pair
cNLS	Classical NLS
dB	Decibels
DNA	Deoxyribonucleic acid
GWAS	Genome-wide association study
HL	Hearing loss
Imp-α	Importin-α
Imp-β	Importin-β
KPNA7	Karyopherin subunit α7
LOD	Logarithm of odds
MAF	Minor allele frequency
ncNLS	Non-classical NLS
NL	Newfoundland and Labrador
NLS	Nuclear localization signal
OPG	Osteoprotegerin
OTSC	Otosclerosis genes/loci
PREMBA	Preimplantation embryo arrest
PTHrP	Parathyroid hormone-related protein
RANK	Receptor activator of nuclear factor *K*β
RANKL	Receptor activator of nuclear factor *K*β ligand
SNP	Single-nucleotide polymorphism
VUS	Variant of uncertain significance
WES	Whole exome sequencing
