# Supplementary material for: Heterozygous Nonsense Mutation in the Nuclear Transport Factor KPNA7, a Maternal Factor Active in Embryonic Tissues, Causes Autosomal Dominant Otosclerosis
Source: Int J Mol Sci. 2026 May 30;27(11):4985. doi: 10.3390/ijms27114985 (PMC13256754; doi:10.3390/ijms27114985)
Supplement: Supplementary file 1 [file ijms-27-04985-s001.zip › ijms-4252892-supplementary.pdf]

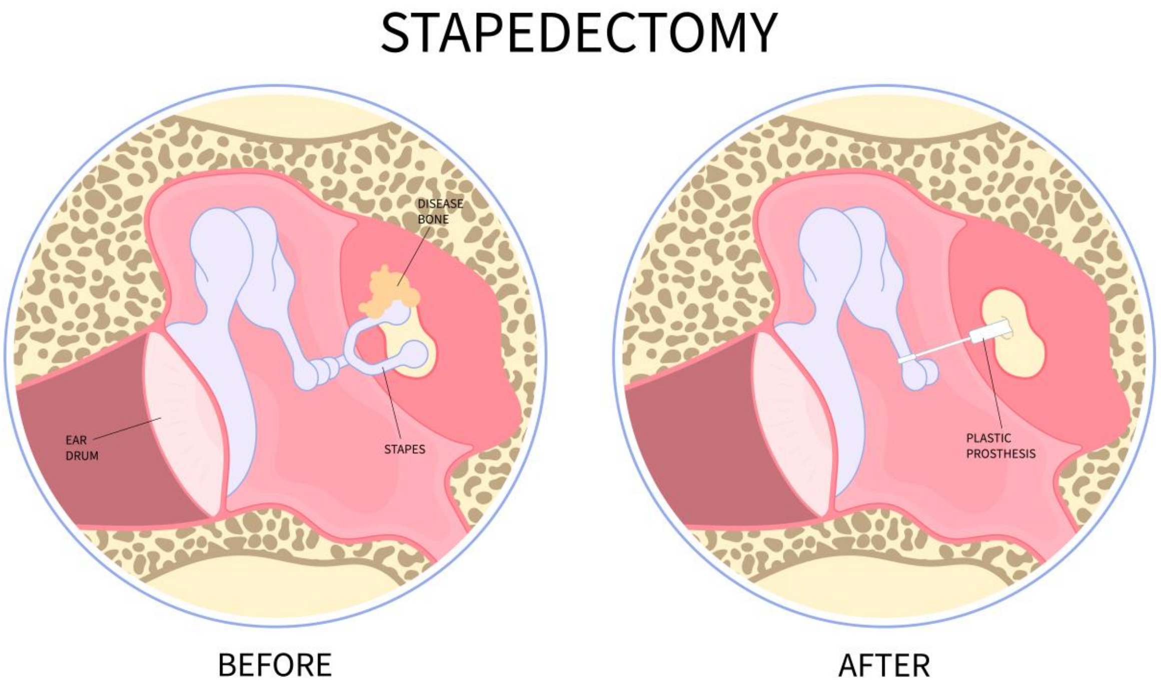

Figure S1. Stapedectomy diagram showing sclerotic lesion on the stapes footplate in the middle ear before stapedectomy surgery, and plastic prothesis replacement after surgery that, in most cases, restores hearing.

Note. From *Meniere's syndrome with stapedectomy surgical for hearing loss and balance problem* [vector illustration], by Pepermpron, 2022, Shutterstock (https://www.shutterstock.com/image-vector/menieres-syndrome-stapedectomy-surgical-hearing-loss-2215745527?trackingId=9128edeb-a02d-4155-bd08-ccd52354a49f&listId=searchResults). Copyright 2022 by Pepermpron. Reprinted with permission.

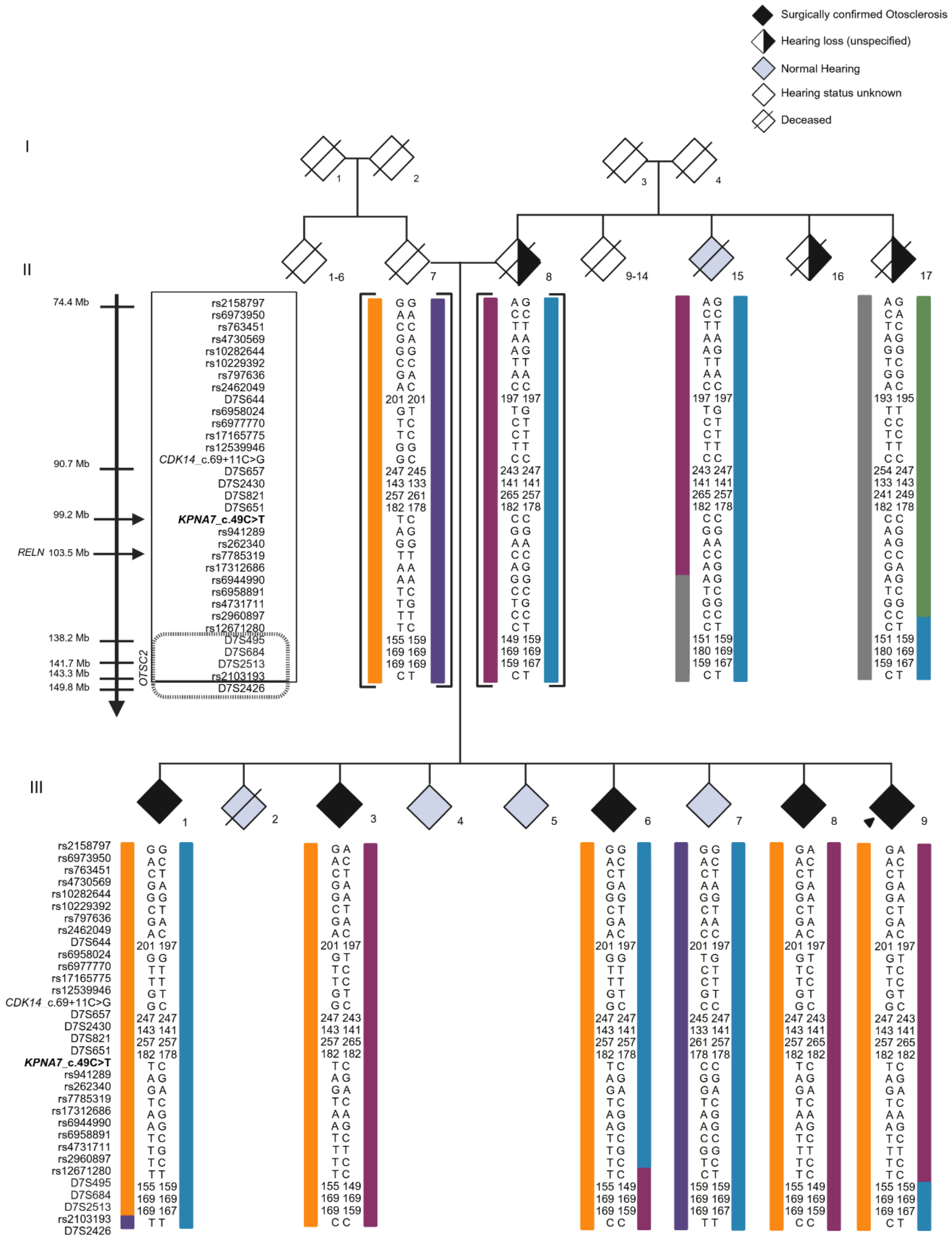

Figure S2. Expanded haplotypes showing overlap of the KPNA7 disease-associated haplotype and the close proximity (within 4.3 Mb) of RELN, an otosclerosis-associated gene. Created in BioRender. BENTEAU, T. (https://BioRender.com/xwjutqk) is licensed under CC BY 4.0.

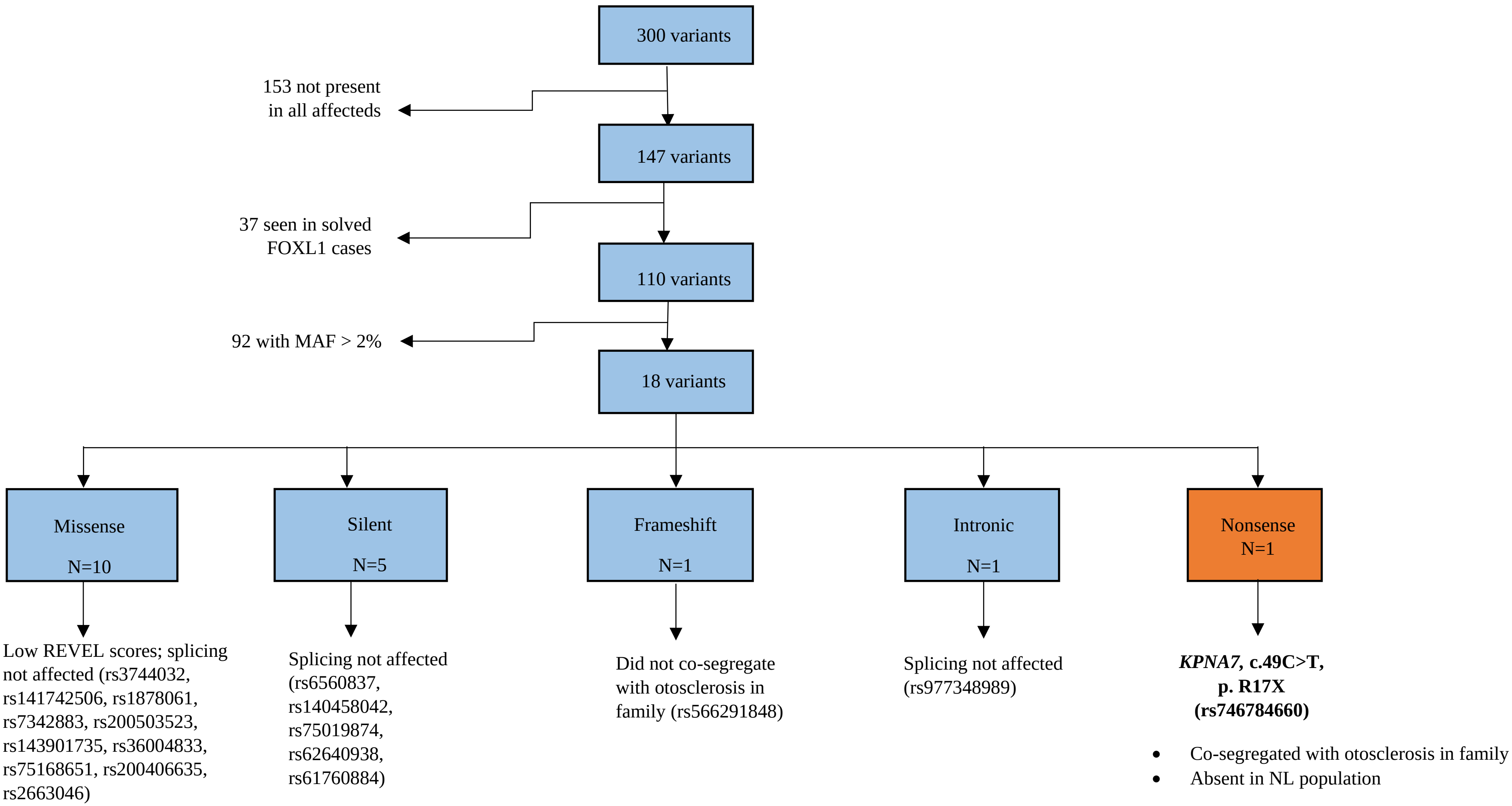

Figure S3. Flowchart depicting WES variant filtering pipeline
Under a dominant mode of inheritance, the maximum theoretical LOD score = 1.7, while linkage analysis yielded a maximum LOD = 1.4 at each of five distinct genomic loci. WES across these five regions revealed >1600 variants but removal of variants also found in two unaffected controls left a total of 300 variants. Upon further pipeline filtering, only one variant remained, KPNA7, c.49C>T.

Table S1. WES variants that passed filtering pipeline with MAF <2%

Gene	Cytogenic location	Variant	SNP ID	SNP status	Splice prediction	REVEL score
ACOX1	17q25.1	c.301G>A, p. G101S	rs3744032	Missense	N/A	0.052
ADAD2	16q24.1	c.1688C>T, p. T563I	rs141742506	Missense	N/A	0.353
CD300E	17q25.1	c.472G>A. p. G158R	rs1878061	Missense	N/A	0.035
EVPL	17q25.1	c.5440C>T, p. P1814S	rs7342883	Missense	N/A	0.061
FRMPD2	10q11.22	c.2735G>A, p. G912E	rs200503523	Missense	N/A	0.036
QRICH2	17q25.1	c.1808 A>G, p. H603R	rs143901735	Missense	N/A	0
SLC13A4	7q33	c.1351C>T, p. P451S	rs36004833	Missense	N/A	0.098
SPAM1	7q31.32	c.1352 A>T, p. D451V	rs75168651	Missense	N/A	0.034
TNRC6C	17q25.3	c.1706 C>T, p. P569L	rs200406635	Missense	N/A	0.181
WDFY4	10q11.23	c.7580G>A, p. S2527N	rs2663046	Missense	N/A	0.014
DIP2C	10p15.3	c.1248 G>A, p. P416	rs6560837	Silent	NI	--
EVPL	17q25.1	c.1347 C>T, p. V449	rs140458042	Silent	NI	--
EVPL	17q25.1	c.3675 C>T, p. S1225	rs75019874	Silent	NI	--
MEAK7	16q24.1	c.1071C>T, p. N357	rs62640938	Silent	NI	--
RNF157	17q25.1	c.186T>C, p. F62	rs61760884	Silent	NI	--
CDK14	7q21.13	c.69+11C>G	rs977348989	Intronic	NI	--
MTNAP1	17q25.1	c.1578del, p. S527Pfs*5	rs566291848	Frameshift	NI	--
KPNA7	7q22.1	c.49C>T, p. R17X	rs746784660	Nonsense	NI	--

Note: REVEL scores >0.5 were considered pathogenic [52]. N/A = Not applicable; NI = No impact
